# Opportunist Coinfections by Nontuberculous Mycobacteria and Fungi in Immunocompromised Patients

**DOI:** 10.3390/antibiotics9110771

**Published:** 2020-11-02

**Authors:** Ines Joao, Helena Bujdáková, Luisa Jordao

**Affiliations:** 1 National Institute of Health Doutor Ricardo Jorge, 1649-016 Lisboa, Portugal; ines.joao@insa.min-saude.pt; 2 Department of Microbiology and Virology, Faculty of Natural Sciences, Comenius University in Bratislava, 842 15 Bratislava, Slovakia

**Keywords:** Nontuberculous mycobacteria (NTM), coinfection, *Aspergillus*, *Histoplasma capsulatum*, *Cryptococcus neoformans*, HIV/AIDS, immunosuppression, opportunistic infections

## Abstract

Nontuberculous mycobacteria (NTM) and many fungal species (spp.) are commonly associated with opportunistic infections (OPIs) in immunocompromised individuals. Moreover, occurrence of concomitant infection by NTM (mainly spp. of *Mycobacterium avium* complex and *Mycobacterium abscessus* complex) and fungal spp. (mainly, *Aspergillus fumigatus*, *Histoplasma capsulatum* and *Cryptococcus neoformans*) is very challenging and is associated with poor patient prognosis. The most frequent clinical symptoms for coinfection and infection by single agents (fungi or NTM) are similar. For this reason, the accurate identification of the aetiological agent(s) is crucial to select the best treatment approach. Despite the significance of this topic it has not been sufficiently addressed in the literature. This review aims at summarizing case reports and studies on NTM and fungi coinfection during the last 20 years. In addition, it briefly characterizes OPIs and coinfection, describes key features of opportunistic pathogens (e.g., NTM and fungi) and human host predisposing conditions to OPIs onset and outcome. The review could interest a wide spectrum of audiences, including medical doctors and scientists, to improve awareness of these infections, leading to early identification in clinical settings and increasing research in the field. Improved diagnosis and availability of therapeutic options might contribute to improve the prognosis of patients’ survival.

## 1. Introduction

Infectious diseases are still a major burden worldwide, causing critical outbreaks such as corona virus disease (COVID-19) and being a persistent cause of mortality, especially in low-income countries. Malaria, HIV/AIDS (Human Immunodeficiency Virus/ Acquired Immune Deficiency Syndrome), lower respiratory tract infections including tuberculosis and diarrheal diseases, account for one-eighth of the total deaths worldwide. In developed countries, cardiovascular diseases are the major cause of death, but infectious diseases are still a huge challenge. In this settings, infectious diseases contribute significantly to morbidity and increased health costs, mainly through chronic infectious and healthcare-associated diseases [1].

Nontuberculous mycobacteria (NTM) disease is generally non-notifiable [2,3], which hinders the correct assessment of its significance [4]. However, it is consensual that an ongoing growth in incidence and prevalence has been observed [5]. Given the lack of mandatory reporting, data are limited to case reviews and research studies, thus incidence and prevalence may be far greater than estimated. To overcome this issue, further prevalence studies and implementation of systematic tracking of NTM diseases using a reporting system are advisable. Despite this drawback, there is an increased body of knowledge on NTM due to the effort of scientific and medical communities, as well as organized study groups such as the European Society of Clinical Microbiology and Infectious Diseases (ESCMID) Study Group for Mycobacterial Infection [6] and NTM- network [7].

Fungal infections are common in humans, and the majority of fungi causing disease are opportunistic pathogens [8]. The scope of current knowledge on fungi is very extensive and continuously updated, not only with basic science findings but also with data collected in clinical setting (e.g., mapping occurrence, prevalence, spread and treatment of fungal disease). This information is summarized by working groups established within the International Society for Medical Mycology [9] as well as the ESCMID study group for fungi and fungal diseases [10]. In spite of this, interaction between fungi and bacteria lacks mandatory reporting, published data being collected on a voluntary basis. Human pathogenic fungi are classified into two groups: the commensals (*Candida* spp.) and the environmental pathogens (*Cryptococcus neoformans*, *Cryptococcus gattii*, *Aspergillus fumigatus*, and thermally dimorphic fungi) [11]. Fungi have frequently caused diseases in immunocompromised patients with HIV, cancer, diabetes and patients treated with immunosuppressive drugs [12,13,14,15,16,17]. In a separate category are healthy individuals with flaws in the immune system due to age (children and elderly), antibiotic treatment or pregnancy [18,19,20,21]. In addition to common diseases, fungi can cause very serious life-threatening infections [14,16,22,23].

Two main concerns are underlined regarding fungal infections: (i) increased rates of multiresistant fungi and (ii) strong potential to colonize host and participate in polymicrobial infections, mainly with bacteria.

Although a great deal of information is available concerning fungi and NTM alone, only little is known about coparticipation of NTM and fungi in human infections. Here, we attempt to fill the gaps, summarizing the clinical significance of coinfection by two groups of opportunistic pathogens (NTM and fungi) from different kingdoms that are able to cooperate and significantly affect the disease course and treatment.

## 2. Opportunistic Infections and Coinfection

Generally, an opportunistic infection occurs when loss of established innate or adaptive immune responses allow none-strictly pathogenic organisms to infect a host. These infections are a major cause of morbidity and mortality among immunocompromised individuals. The type and severity of the immune defect determines the profile of the potential aetiological agent and the opportunistic infection [24]. Taking into account an infectious trait of NTM and major fungal pathogens, as well as the already mentioned definition of OPIs, there are two different points of view. While Mycobacterium is associated with host colonization and/or infection, some fungal spp. are members of the common human microbiome and others are environmental pathogens. For example, *Candida albicans* colonizes over 50% of the population without any symptoms. It can be present in the skin, oral cavity, intestine, upper respiratory tract and female reproductive tract [25,26,27,28,29,30]. However, under optimal circumstances, this yeast is able to switch from a safe commensal to a dangerous pathogen able to cause infections whose treatment is very challenging [31]. A distinct situation is the case of airborne microorganisms, such as filamentous fungi (Aspergillus, Rhizopus, Mucor, etc.) and other already mentioned environmental pathogens (*H. capsulatum*, *C. neoformans*) colonizing the host via the upper respiratory tract [14,16,17].

Coinfection occurs when pathogens (e.g., viruses, bacteria, fungi, parasites) co-occur within an individual [32], most often with a chronic infection [33]. Parasite coinfections [34] and HIV/*Mycobacterium tuberculosis* coinfection [35,36] are huge burdens for human health. Accurate knowledge of coinfection prevalence is missing, but it is estimated that at least one-sixth of the human population is affected by this condition. In addition, a negative effect of coinfection on human health is more frequent than no effect or positive effect [32]. The fact that individuals with poor health are more prone to coinfection might contribute to this outcome. Other contributing factors are synergistic relations between pathogens by modulation of the host immune system, resource competition, interfering with the performance of diagnostic tests, drug-drug interaction and emergence of drug resistance [32,37,38]. Coinfection can dramatically modify infection dynamics through ecological mechanisms such as convalescence and disease-induced mortality, and an effort has been made to develop models to evaluate the risk assessment [33,39].

Although in this review, the focus is on coinfection by opportunistic pathogens from the bacteria kingdom (NTM) and fungi kingdom, there are many reports of coinfection by different representatives of viruses, bacteria, fungi and parasites. Among them, coinfection in HIV-patients is very frequent. For example, yeasts of the genus Candida or the fungus *Pneumocystis jirovecii* occur frequently and can cause life-threatening pneumonia in these patients [22,23]. HIV infection is also strongly associated with a higher prevalence of chronic human papillomavirus, resulting in increased risk for anal cancer [40]. In HIV-Leishmania coinfected patients, visceral leishmaniosis can accelerate disease, decreasing the likelihood of patient survival [41]. *C. neoformans* and *H. capsulatum* are the only agents that have been isolated, particularly from immunocompromised individuals [14,16]. Their participation in coinfection is very rare, but Nunez and colleagues reported simultaneous infections in an HIV-negative 69-year-old man with chronic obstructive pulmonary disease and type II diabetes mellitus [42]. Invasive pulmonary aspergillosis is a fungal disease strongly associated with immunocompromised patients [43]. Invasive pulmonary aspergillosis was reported in immunocompetent patients severely infected either with influenza B or influenza A H1N1 virus [44]. *A. fumigatus* and *Pseudomonas aeruginosa* coinfection in patients with cystic fibrosis is often observed [45]. Concomitant aspergillosis and mucormycosis is very rare, but this combined disease was described in a 17-year-old girl with subsequent treatment of a recurrent glioma [46]. Coinfections by multiple NTM species have been reported mainly as case reports. *Mycobacterium malmoense* and *Mycobacterium chimaera* in a kidney transplanted patient [47], pleural effusion by *Mycobacterium fortuitum* and *Mycobacterium mageritense* [48], mixed pulmonary infection by *Mycobacterium avium* or *Mycobacterium intracellulare* (MAC) and *M. abscessus* or *Mycobacterium massiliense* (MABC) [49,50] are examples of case reports. Furthermore, it has been recently described that coinfection by multiple NTM spp. could exacerbate the symptoms of pulmonary lung disease by these pathogens [51]. Finally, coronavirus infection together with influenza A virus or different bacteria, were summarized in the work of Cheng-Lai and colleagues [52]. The difficulties with treatment of all above mentioned coinfections underline the fact that the majority of them resulted in mortality.

## 3. The Aetiological Agents: NTM and Fungi

NTM and fungi are ubiquitous environmental microorganisms potentially pathogenic to humans. NTM are a heterogeneous group formed by almost 200 species [53], with only two strict pathogens: *Mycobacterium marinum* and *Mycobacterium ulcerans*, which are responsible for the fish tank granuloma and buruli ulcer, respectively [54,55,56]. Although the majority are nonpathogenic to humans, NTM are recognized aetiological agents of OPIs, in particular species belonging to the MAC and the MABC. NTM infections are phenotypically diverse, manifesting themselves as a large spectrum of diseases affecting nearly all organs [57]. Being ubiquitous in nature, NTM share with humans and animals a wide variety of habitats and niches leading to high rates of human–pathogen contact [58].

The majority of fungi associated with human diseases are able to switch from a transient environmental contaminant or a saprophytic life-style to a parasitic life-style [11,59,60]. Generally, the yeast *C. albicans* and the mold *A. fumigatus* are the most frequent pathogens causing invasive mycoses, candidaemia being the most common infection [61,62]. Koehler and colleagues provided a systematic review (from January 2000 to February 2019) on candidaemia morbidity and mortality in Europe [63]. From a retrospective study, they extrapolated from approximately 79 cases of candidaemia per day why 29 patients out of 25 may be fatal at day 30. Environmental pathogen *C. neoformans* is associated with invasive infections of the central nervous system (CNS), mainly in HIV/AIDS patients but, like *C. gattii*, it can also cause pulmonary cryptococcosis [64]. CNS infection caused by molds (*A. fumigatus*) and other aspergilli typically register mortality rates between 50–100%. Aspergillosis usually occurs after manifestation of pulmonary disease in immunocompromised and organ transplant patients [17]. Aspergilli rarely cause fungaemia, with the exception of Fusarium spp. [65,66].

The transmission of NTMs can occur through an environmental source or clinical settings to the patient [67]. Person to person transmission has been suggested for *M. abscessus* but has not been fully demonstrated [68,69,70]. This ubiquity and exposure poses an enormous challenge for clinicians, since isolation of NTM from a clinical sample may only indicate a transient or persistent colonization, or an infection [71]. Aspergillus, Cryptococcus and Histoplasma, as typical environmental fungi, are not transmissible between humans. Human infection occurs by inhalation of spores or the yeast form via the respiratory tract [11]. Therefore, ventilation or air conditioning with intensive aerosol production facilitates these pathogens spreading [72]. In contrast to the mentioned fungal spp., as well as to NTM, certain Candida spp. can be transmitted by direct contact with medical staff or other patients, making it difficult to determine if an infection has an endogenous or exogenous origin [60,73].

Despite being globally disseminated, NTM spp. exhibit uneven geographic distribution, which can be attributed to the environmental nature of the genus. With the exception of Japan, MAC is the most common species in Asia, in most of western countries (European Union (EU) included) and African countries. In most western countries and in the EU, *Mycobacterium gordonae* and *Mycobacterium xenopi* [74], together with *M. intracellulare* and the rapidly growing *M. fortuitum,* are the next most common NTM spp. [3]. In the United States of America (USA), *Mycobacterium kansasii* and *M. abscessus* follow MAC spp. [5], and in Asia, *M. intracellulare* is more common than *M. avium* among MAC spp, with the known exception of northern Japan, in which species from MABC are the second most frequent [75]. Common species in Africa are *Mycobacterium sherrisii*, and species related to occupational or geographic incidence, such as *M. kansasii* in mining workers and *Mycobacteriu lentiflavum* in Zambia, respectively [75]. The relatively organized distribution portrayed above is punctuated by several divergences reported in the literature [76,77,78]. The uneven geographical spread of NTM represents a challenge in terms of pathogenesis knowledge, diffusion and infectious disease management requiring a wide array of distinct therapeutic approaches. NTM growth rate is another factor that influences the therapeutic scheme. NTM are divided into rapidly growing mycobacteria (RGM, e.g., *M. fortuitum*, MABC: *Mycobacterium chelonae* and *M. abscessus*), and slowly growing mycobacteria (SGM, e.g., MAC, *M. xenopi* and *M. kansasii*) exhibiting differential epidemiology of infection. Usually, RGM infections are mostly cutaneous and osteoarticular, whereas SGM infections are located in lungs and lymph nodes [79], implicating a direct correlation between growth rate and virulence.

Despite the global distribution of *H. capsulatum*, its variety capsulatum is preferentially endemic in the soils of specific American regions, sub-Saharan Africa and South-East Asia [80,81]. *C. neoformans*, *H. capsulatum*, and *A. fumigatus* are common environmental pathogens distributed worldwide [11]. Another example of yeasts with a worldwide distribution is Candida. It has been already mentioned that *C. albicans* is the predominant spp. However, an increased proportion of *Candida* spp. other than *C. albicans* is the current trend, as illustrated by candidaemia cases by non-*C. albicans* spp. observed worldwide [63,82]. Candidaemia is strongly associated with patient catheterization (49.7%), with *C. parapsilosis* being the most common causative agent of candidiasis in this population (58%) [83]. In USA, *C. albicans* remains the leading aetiological agent of invasive candidiasis, followed by *C. glabrata* [84]. In EU, the situation in adults is similar, with the prevalence of *C. albicans* highest (>50%), followed by *C. glabrata* (13–22%) then by *C. parapsilosis* (about 15%) depending on the hospital or region monitored [66,85]. In children and teenagers, *C. albicans* (>50%) is also the predominant yeast, followed by *C. parapsilosis* (28%). A higher proportion of candidemia caused by *C. albicans* was observed among newborns (60.2%), with highest rates of *C. parapsilosis* being observed among infants (42%) [86].

## 4. The Human Host: Predisposing Conditions for NTM, Fungal Infections and Coinfections

Some predisposing conditions of human hosts to develop NTM or fungal disease are similar, but some of them differ with respect to fungal spp. In general, NTM and invasive fungal infections mostly occur in immunocompromised patients. For instance, histoplasmosis or cryptococcosis are strongly associated with acquired immunodeficiency [87,88]. Nevertheless, predisposing nonimmunological conditions can significantly contribute to the development of infections caused by Candida spp., namely diabetes, pregnancy and age, but also burn-patients and healthcare associated infections [15,18,21,89,90]. Although NTM can cause infection at a wide variety of body sites (e.g., skin, soft tissues and lymphatic nodes), lung infections are the most frequent. Patients with structural lung diseases such as chronic obstructive pulmonary disease (COPD), bronchiectasis, pneumoconiosis, prior tuberculosis and pulmonary alveolar proteinosis, are more prone to NTM infections [5]. Rheumatoid arthritis, cystic fibrosis (CF), gastroesophageal motility disorders and other chronic diseases with pulmonary manifestations including lung malignancies, are other factors that can predispose an individual to NTM pulmonary infections [5,91,92]. Pre-existing pulmonary diseases are also a predisposing factor for opportunistic pulmonary infections by Aspergillus spp. the severity of the clinic manifestations being conditioned by the host immune status [93]. The existence of underlying pulmonary disease and immune disorders favor infections by both opportunistic pathogens fungi and NTM [71].

Patients with immune suppression, either primary or acquired, are more susceptible to NTM infections, namely to disseminated NTM infections [94]. Disseminated infections by MAC in AIDS patients brought NTM to the spotlight back in the 1980s [95]. Until the introduction of antiretroviral therapy (ART) in the 1990s, OPIs by mycobacteria and fungi, among other pathogens, were a major cause of mortality and morbidity in HIV-infected patients. In order to overcome this problem, treatment guidelines for OPIs were implemented and further updated over the years [96,97].

Primary immune suppression, such as Mendelian susceptibility to mycobacterial disease mediated by mutations in IFN-γ and IL-12 receptors, deformities in the STAT1, IKKKG, GATA2 genes and production of autoantibodies to IFN-γ (interfering with STAT1 phosphorylation and IL-12 production) [98] increase the risk of disseminated NTM [68,94], Cocccidioides and Histoplasma infections [99]. Additionally, a STAT1-gain-of-function has been described to cause Th17 deficiency resulting in chronic mucocutaneous candidiasis (CMC) [100]. Similarly, impairment of STAT3 signaling affecting IL-6, IL-21 and IL-23 pathways, and defective generation of Th17 responses, are associated with Hyper-IgE Syndrome. Patients with this syndrome are vulnerable to CMC, but also to Histoplasma, Coccidioides, and Cryptococcus [101,102,103]. Caspase domain containing protein 9 (CARD9) has a key role in regulation, being a central regulator of innate immunity expressed in neutrophils, macrophages and dendritic cells. The correlation between autosomal recessive CARD9 deficiency and susceptibility to invasive fungal infections, including Candida spp. or *A. fumigatus*, has been described [104], but this deficiency is also associated with CMC [105]. Chronic pulmonary aspergillosis was observed to be a risk in the variants of genes coding for cytokines (IL1B, IL1RN, IL15), pattern recognition receptors of TLR1, CLEC7A (Dectin-1), DENND1B and VEGFA genes [106,107].

Acquired immunosuppression induced by drugs such as corticosteroids, chemotherapeutics to treat cancer, immunosuppressive drugs to prevent rejection after organ transplant, TNF-α antagonists and other biologic chemotherapeutics to treat inflammatory conditions, are other factors that could promote OPIs [68,71,94]. Macrophages play a key role in mycobacterial pathogenesis. Mycobacteria-infected macrophages trigger CD4^+^ T-helper cell pathways through IL-12 and IFN-γ that activate macrophages promoting pathogen clearance [108]. This explains why low CD4^+^ counts in HIV-patients (a determinant for initiation of ART) and the presence of circulating anti-IFN-γ promote OPIs by NTM. On the other hand, the proinflammatory cytokine, TNF-α, plays a key role in granuloma formation and maintenance, controlling mycobacteria spread ([109,110] for recent review on host immune response to NTM). A similar scenario was observed for histoplasmosis, with milder cases of the disease being reported in patients with cell-mediated immunodeficiency treated with TNF-α inhibitors [111]. TNF-α, being a proinflammatory cytokine, stimulates the production of other cytokines such as IL-2 and IL-12, important for the host immune response. TNF-α stimulates IL-12 and IFN-γ production, promoting a Th- mediated response to *C. neoformans* lung infection [112]. In Candida biofilms, resistance to phagocytosis was associated with a modified cytokine profile, especially by TNF-α downregulation [113]. This observation was confirmed in a study with patients manifesting recurrent vulvovaginal candidiasis, in which TNF-α production triggered by *C. albicans* hyphae was significantly higher compared to the control group [114]. Fungal cell wall components play a crucial role in the interaction with host cells. The β-1,3 –glucan, a basal component of the fungal cell wall, is specifically recognized by a pattern recognition-receptor named Dectin-1. This cell wall component plays an important role in the adaptive immune response to *A. fumigatus* infection mediated by increased production of CXCL1, CXCL2, and TNF-α by bone marrow-derived macrophages [115].

## 5. Coinfection by NTM and Fungi

Concomitant isolation of mycobacteria and fungi from patients’ respiratory samples is a complex situation that could lead to misdiagnosis and inadequate therapy. The majority of reports concerning concomitant NTM and fungal infections, during mainly the last 20 years, is presented in Table 1 although ancient reports are also quoted in this section. First, as previously described in this manuscript, being opportunistic pathogens, isolation of NTM and fungi does not necessarily indicate an infection, a colonization scenario being also possible. This duality is present in the literature reports on NTM and fungi spp. isolation in patients’ samples. The case reports describe infection cases [116,117,118,119,120,121,122,123,124,125,126], but for several prospective and retrospective studies either it is not clear, or both infection and colonization are present [71,120,127,128,129,130,131,132]. There is evidence that coinfection by fungal and mycobacterial spp. worsens the prognosis of patients with lung disease, an increase in morbidity and mortality being observed for both *M. tuberculosis* and NTM [127,128,133,134]. For patients with pulmonary tuberculosis, *A. fumigatus*, *Aspergillus niger*, *H. capsulatum*, and *C. neoformans* are the main aetiological agents of infection [129]. Although for coinfection with NTM, all the previously mentioned fungi were identified, *Aspergillus* spp. being predominant and other fungi such as *Talaromyces marneffei*, and Candida spp. also described (Table 1).

Retrospective and prospective studies, mainly monocentric, have been designed to answer different questions related to NTM/ fungi coinfection. Providing evidence that these opportunistic pathogens could be isolated from sputum is either the most frequent, or a starting point for others [71,127,128,130,131,132,135,136,137,138,139]. Comparing the results from different studies is not straightforward due to differences in study designs and studied populations. For instance, Samayoa and colleagues aimed at evaluating the incidence and mortality of tuberculosis, histoplasmosis and cryptococcosis in HIV patients, with the identification of NTM and fungi coinfection as a side result [131]. An attempt to identify risk factors for coinfection in patients with specific clinical conditions was made [132,138,139]. COPD, pulmonary *M. intracellulare* disease and systemic corticosteroid use were identified as main risk factors for coinfection with Aspergillus [138]. Although colonization of pre-existing pulmonary lesions (cavities) by fungi, namely Aspergillus spp., is well-documented [130,140,141,142], NTM-fungi coinfection is rare [71,137,140] and the clinical relevance of its coisolation is species-dependent. Isolation of nonfumigatus Aspergillus strains (e.g., *A. niger*, *Aspergillus terreus*, *Aspergillus flavus*) from respiratory samples was most often associated with colonization [127]. In cystic fibrosis patients *A. fumigatus* and *Scedosporium* sp. are regarded as threats.

*M. monacense*, *M. kansasii*, *M. xenopi*, *M. intermedium* and *M. fortuitum* were the non-MAC or non-MABC NTM spp. responsible for coinfection with fungi in immunosuppressed patients. Coinfection of *M. intermedium* and *M. fortuitum* with *T. marneffei* and *Aspergillus* spp., respectively, were reported in patients with high titers of anti-IFN-γ antibodies [143,144].

A severe case of pneumonia, with isolation of *Candida glabrata* among other microorganisms from bronchoalveolar lavage fluid (BALF), and *M. monacense* from hemoculture, was reported by Yuan and colleagues [125]. *M. monacense*, an NTM was newly identified in 2006 [145], with few reports of isolation from human samples and clinical significance not clearly understood. This last feature is shared with *C. glabrata* in which isolation from BALF was transitory suggesting that in this case cross-contamination, transient infection or colonization are plausible scenarios [125]. The relevance of *Candida* spp. is still unclear [146] despite frequent reports of oral candidiasis in AIDS patients with disseminated infections [147,148]. The under-valorization of Candida isolation from respiratory samples was illustrated in a case report with concomitant isolation of *M. kansasii* from sputum and the prescription of a therapeutic scheme directed exclusively to MNT [149].

MAC *(M. avium* and *M. intracellulare*) and MABC (*M. abscessus* and *M. massiliense*) were considered more prone to cause infection, whereas *M. chelonae*, *M. gordonae* and *M. fortuitum* were more likely to be regarded as colonizers [71]. Curiously, a coinfection by *M. chelonae* and *Scedosporium apiospermum* was reported in an immunocompetent individual [126]. The patient had a history of diabetes (a predisposing condition for NTM infection) and hyperlipidemia controlled by medication and, after a skin antibiotic test as part of a prechirurgical procedure, presented skin lesions compatible with an infectious process. The two opportunistic pathogens were isolated, but it was not clear how the patient was infected. All the other case reports summarized in Table 1 occurred in patients with at least one predisposing condition for NTM infection. Coinfection by *M. avium* and *A. fumigatus* was described in a 65-year-old female with previously diagnosed COPD, productive cough, fatigue and weight loss for three months [130].

In the past, other opportunistic fungal infections started by being neglected and then included in the definition of a clinical situation. This was the case of histoplasmosis that only in 1985 was taken into account for the definition of AIDS by the Centers for Diseases Control and Prevention (CDC) [150]. Curiously, all but one of the *H. capsulatum*-NTM coinfections reported in Table 1 are cases of disseminated infections in AIDS patients, *M. avium* being the predominant NTM [136,151,152,153,154,155] and *M. sherrisii* the other NTM [156]. In Table 1, several cases of coinfection by MAC and *H. capsulatum* are presented, whereas other pathogens such as *E.coli* [152], herpes virus and *Pneumocystis jirovecii* [153] were isolated. In two case reports of non-HIV patients, in addition to NTM-fungi coinfection either cytomegalovirus (CMV) or varicella zoster virus were involved [119,122]. Triple coinfections with CMV, MAC and Aspergillus, or varicella zoster virus (VZV), *M. abscessus* and *Cryptococcus,* were reported in immunosuppressed patients by cancer or autoimmune disease with IFN-γ antibodies production, respectively [122]. The virulence of the NTM and the imbalance of the host immune system certainly played a role in this outcome.

## 6. Treatment: Major Challenges and Future Perspectives

NTMs’ susceptibility to standard antituberculosis (TB) drugs displays significant heterogeneity, standard anti-TB drug regimens being ineffective on NTM disease treatment [158]. Anti-NTM regimens are long (at least 18 months), and a 12 months period with sputum-negative results is required to confirm the cure [159,160]. Sputum-conversion, from a positive to negative finding of bacteria, is often difficult to achieve in NTM cases, especially for infection with macrolide-resistant NTM spp. [161]. Despite the existence of specific guidelines, based on the nature of infecting mycobacteria and requiring spp. identification, treatment of NTM disease is mostly empirical and not entirely successful [161]. The treatment of NTM diseases usually involves the use of macrolides and injectable aminoglycosides. Resection surgery of affected organ(s) in the case of patients that do not respond to antibiotic based treatments, is also an option [162].

Drug susceptibility tests (DSTs) for NTM are challenging and controversial because of inconsistency between in vitro results and clinical outcomes [161]. Macrolides are an exception exhibiting a good correlation between in vitro susceptibility results and clinical response. For this reason, macrolides are the pillar of current NTM infection treatment protocols [163,164], clarithromycin or azithromycin being the usual options [165]. For patients with nonsevere MAC-pulmonary disease, the recommended initial therapy consists of a clarithromycin or azithromycin, ethambutol (ETB) and rifampicin (RIF) regimen. In the case of severe conditions, injectable amikacin or streptomycin is advised [159,160]. For clarithromycin-resistant MAC infections a regimen including RIF, ETB, either isoniazid (INH) or a quinolone, and an injectable aminoglycoside should be adopted [160].

The treatment of *M. abscessus* lung disease faces significant challenges due to intrinsic antibiotic resistance [164], oral macrolides combined with two parenteral drugs being the medication of choice [161]. *M. massiliense,* one of the three subspecies of MABC, harbors a partial erm41 gene deletion preventing inducible macrolide resistance and leading to higher rates of successful treatment with macrolide-based antibiotic regimens compared to infections by *M. abscessus* (functional erm41 gene) [166,167]. Therefore, the correct identification of these subspecies, and precise diagnosis, are crucial for the treatment of infected patients [166,167].

*M. kansasii* has similar disease/ pathology presentation to pulmonary TB, a regimen with RIF, ETB, INH being recommended for drug-sensitive *M. kansasii* [159]. Alternatively, BTS (2017) recommends INH combined with pyridoxine, as an alternative to macrolides [160]. A three-drug regimen is recommended for rifampin-resistant strains including azithromycin, ETB and a fluoroquinolone [159].

Pulmonary disease due to *M. malmoense* is a challenge to treat [159]. The use of INH, RIF, ETB with and without quinolones and macrolides [159] is recommended. However, the BTS guidelines recommend a minimum three-drug regimen including RIF, ETB and a macrolide as a daily treatment, in the case of nonsevere disease, and the use of additional injectable drugs, such as amikacin or streptomycin, for severe cases [160].

In individuals with *M. xenopi* pulmonary disease, improved outcomes may be achieved using a four-drug antibiotic regimen comprising RIF, ETB and a macrolide (clarithromycin or azithromycin), with either a quinolone or INH. For severe NTM diseases, injectable or nebulized amikacin is added to the above regimen for up to three months [160].

In severely disseminated or recalcitrant disease, and in cases of macrolide-resistant MAC, second-line drugs are extremely important. Aminoglycosides (e.g., amikacin or streptomycin) are most often used in such cases [5]. Fluoroquinolones, in addition to having significant activity against *M. kansasii*, play a crucial role in cases of RIF-resistant *M. kansasii* [168]. Clofazimine has also shown favorable activity against pulmonary disease [169]. Linezolid has been used with success in the treatment of skin and soft tissue diseases caused by RGM *M. chelonae* and *M. abscessus*.

Fungal infections can be treated with antifungal compounds from several groups, but the selection of the appropriate drug depends on fungal species, type of infection and, in the case of concomitant infection, possible interferences has to be assumed. Taking into account information from Table 1 on distribution of fungal spp. participating in coinfection with NTM, amphotericin B and azoles, namely itraconazole and voriconazole, are the most frequently used drugs for treatment. Amphotericin B is a polyene antibiotic binding to ergosterol, the main sterol of fungal membranes. This interaction causes disruption of the cytoplasmic membrane through the formation of ion channels leading to leakage of monovalent protons and cytoplasmic contents. Additionally, it promotes reactive oxygen species production leading to oxygen depletion [170,171]. Amphotericin B in lipid formulations is preferred over amphotericin B deoxycholate, as it is more pleasant to patients and provides better delivery to organs such as lungs, liver, and spleen [172,173]. Azoles interfere with ergosterol biosynthesis through binding to the cytochrome P450-associated enzyme 14-α-demethylase that converts lanosterol to ergosterol [172,174]. The antifungal drug 5-fluorocytosine is an inhibitor of nucleic acid synthesis [175], being used only in combination with the azole derivative fluconazole in the treatment of cryptococcosis. Amphotericin B, its lipid formulations, and fluconazole are other options for monotherapy [176,177]. NTM/ fungal coinfection is not easy to treat, as amphotericin B causes a wide spectrum of adverse effects including reversible nephrotoxicity and, similarly to azoles, an interference with human cholesterol pathway was observed, including mammalian CYP 450 enzymes, resulting in abnormal profiles of liver enzymes [178]. Although better tolerated, fluconazole is not recommended for treatment of *C. glabrata*, *Candida krusei*, and *Aspergillus* spp. infections due to residual or nonexistent activity. Therefore, other azole derivatives, namely itraconazole and voriconazole, are used in the therapy of pulmonary aspergillosis [122]. Severe histoplasmosis cases requiring patient hospitalization are treated with liposomal amphotericin B and therapy is usually changed to itraconazole after one to two weeks. For nonlife-threatening histoplasmosis cases, itraconazole is the drug of choice [179]. The next groups of antifungal agents are echinocandins, produced by fungi as secondary metabolites. Currently, caspofungin, micafungin and anidulafungin are on the market, being effective against invasive *Candida* infections, and are reserve drugs for infections by *Aspergillus* spp. in monotherapy regimens when amphotericin B is not well tolerated [180,181]. Echinocandins block function of β-(1,3)-D-glucan synthase, the key enzyme in fungal cell wall synthesis, and are inefficient against the capsulated *C. neoformans* [182]. Echinocandins can be also used in combination with azoles and amphotericin B [183].

In the case of coinfection by fungi and NTM, the same drugs are used for treatment. Next, treatments used in coinfection cases will be mentioned in more detail. In a patient with steroid-treated sarcoidosis a coinfection by *A. niger* and MAC was described. Liposomal amphotericin B was selected for aspergillosis treatment but the patient prognosis was poor [117]. In another case of coinfection by *M. avium* and *A. fumigatus*, antifungal itraconazole and antibacillar RIF were administered, but a drug interaction was observed, and RIF was suspended. Later on, itraconazole was replaced by voriconazole, an improvement in patient condition being observed only after six months treatment. The observed drug-drug interaction supports the combination of RIF with voriconazole instead of itraconazole. The analysis of other case reports summarized in Table 1 shows that amphotericin B and/or new triazole derivatives are the drugs of choice to treat concomitant invasive aspergillosis and NTM infection [119]. In the already mentioned case of a 53-year-old man with combined infection of *Cryptococcus*, *M. abscessus*, and VZV [122], amphotericin B together with fluconazole was selected to treat cryptococcosis in combination with amikacin, imipenem, azithromycin and levofloxacin for NTM, and intravenous acyclovir for VZV [122]. In another coinfection case by C. *glabrata*, *M. monocense* and other bacteria, voriconazole was administered together with cefoperazone, sulbactam and linezolid, an improvement in the patient’s conditions being observed after 10-days therapy [125]. In a previously mentioned report by Basso and colleagues, the treatment of a patient with HIV infection and histoplasmosis was described [154]. The regimen was complex including several antifungal drugs over time (intravenous amphotericin B deoxycholate, itraconazole, itraconazole), antiretroviral (tenofovir, lamivudine, efavirenz) and antibacillar drugs (sulfamethoxazole-trimethoprim, clarithromycin, ETB, streptomycin and levofloxacin). The likelihood of drug-drug interaction with occurrence of adverse drug effects increases in such regimens when the patient takes multiple drugs (polypharmacy) [184]. In the case of NTM-fungi coinfection in immunocompromised patients, other factors such as the immune status of the patient, existence of background diseases (e.g., diabetes) and the interaction with other compounds such as anticancer drugs, has to be considered in order to select a successful therapy.

Because NTM diseases require long treatment periods, often leading to severe secondary effects and often disappointing clinical outcomes, there is an urgent demand to discover and develop new, more efficacious anti-NTM conventional drugs [158] and to explore alternative approaches. Several new drugs currently in the drug–discovery pipeline might improve the treatment of NTM diseases, offering additional choices and new drug combination options [158]. For example, bedaquiline, in addition to being promising against several species of RGM and SGM NTMs, may also have potential clinical applications in patients with MAC or MABC lung disease [185,186]. Nevertheless, the repurposing of conventional drugs is also a promising alternative, e.g., chloroquine and primaquine with activity against *M. avium* [187,188]. Tests conducted in mice models with *M. avium* and *M. abscessus* showed promising results for clofazimine administered by inhalation [189]. The same option has been developed for pulmonary aspergillosis, where aerosolized delivery of liposomal amphotericin B, voriconazole or posaconazole is applicable [190,191]. Antimicrobial peptides exhibit a broad-spectrum of antimicrobial activity against bacteria, viruses, parasites and fungi, and could be alternatives for the treatment of mycobacterial infections [192]. Other alternatives include a phage therapy with the use of bacteriophages [193] and host-directed therapies (HDT), which might improve the clinical outcome of NTM therapy and/or reduce the duration of treatment [161]. Natural approaches are also gaining consideration in overcoming antimicrobial resistance in NTMs, especially compounds from plants and venoms [194]. Another alternative are mycogenic metal nanoparticles produced by filamentous fungi with antimicrobial and anti-inflammatory activity [195]. However, conventional therapy is still the most effective to treat immunocompromised patients coinfected with multiple infectious agents.

## 7. Conclusions

This review summarizes information on concomitant infection by NTM, represented mainly by, but not restricted to MAC spp., and pathogenic fungi. Members of MABC, mainly the RGM *M. abscessus* is gaining growing relevance due to intrinsic antibiotic resistance and recently proposed human to human transmission. A panoply of other RGM (e.g., *M. fortuitum*, *M. chelonae*, *M. monassence*) and SGM (e.g., *M. kansasii*, *M. xenopi*, *M. gordonae*, M. sherrisii) have been associated with coinfection.

**Figure 1:**
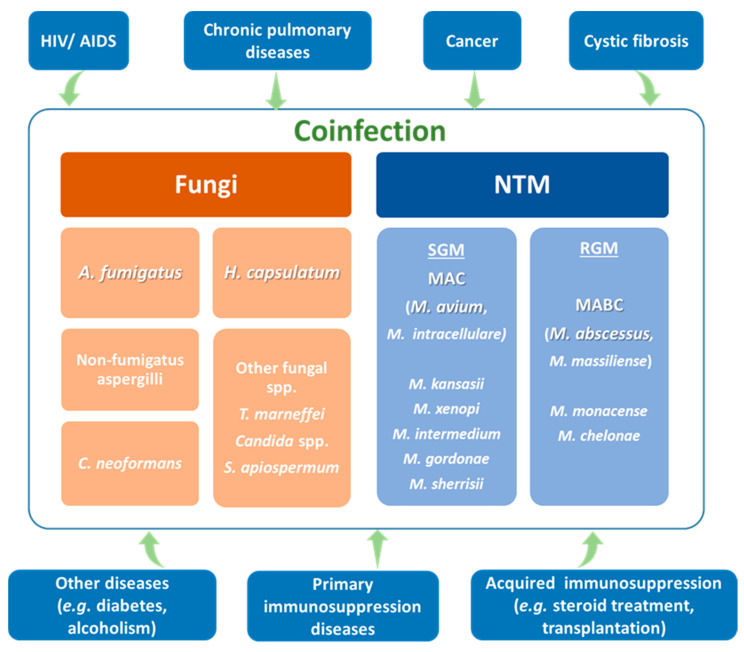
Schematic representation of the most relevant nontuberculous mycobacteria (NTM) and fungal spp. participating in coinfection, and their relation to factors affecting host health status. Rapidly growing mycobacteria (RGM), slowly growing mycobacteria (SGM), MAC (*M. avium* complex), MABC (*M. abscessus* complex).

Figure 1 provides a short summary from literature reports listed in Table 1. Taking into account information from this table, *A. fumigatus,* together with nonfumigatus spp., identified Aspergilli and *H. capsulatum* as the major aetiological agents observed in coinfection with NTM. Fewer reports for *C. neoformans*, *T. marneffei* and Candida spp. were found, suggesting that they play a minor role in those infections. This representation of fungal spp. confirms that air-transmitted fungi named as environmental pathogens play a key role in coinfection with NTM in pulmonary infections. The huge diversity of pathogens involved makes the diagnostic and implementation of effective therapeutics with the currently available chemotherapeutic options quite challenging. This scenario is worsened by the poor immune status of the patients, polypharmacy leading, most likely, to drug-drug interaction, and the appearance of more or less serious side effects. The prognosis of such patients is usually uncertain. However, a prediction of possible complications, taking into account the nature of the patient’s original disease, or an early evaluation of symptoms and correct identification of infectious agents, improves the prognosis of patient’s survival.

## Figures and Tables

**Table 1:** NTM and fungal coinfection.

Underlying Diseases *	Aetiological Agents		Reference	Observations
	NTM	Fungi		
Immunosuppression (steroids treatment)	MAC	*A. niger*	[117]	Case report.
HIV infection	MAC	*Cryptococcus*	[118]	Case report.
Cancer	MAC	*Aspergillus* sp.	[119]	Case report.
Chronic pulmonary infection	MAC	*Aspergillus* sp.	[120]	Case report.
Multiple diseases (oral and colon cancer, hematologic malignancies, connective tissue disease, diabetes, corticosteroid immunosuppression)	*M. kansasii*	T. marneffei**	[121]	Case report.
Autoimmune disease (anti IFN-γ ab)	*M. abscessus*	*Cryptococcus* sp.	[122]	Case report of triple infection.
AIDS (Kaposi sarcoma)	*M. avium intracellulare*	*C. neoformans*	[123]	Case report.
Pulmonary MAC disease	MAC (*M. avium and M. intracellulare*)	*Aspergillus* spp. (*A. fumigatus* and *A. niger*)	[135]	Chronic necrotizing pulmonary aspergillosis (CNPA) as a complication of pulmonary MAC disease.
AIDS	*M. intracellulare*	*T. marneffei*	[124]	Case report.
AIDS	*M. avium*	*H. capsulatum*	[154]	Case report.
Pneumonia	*M. monacense*	*C. glabrata*	[125]	Case report of *M. monacense* was isolated from blood whereas *C. glabrata* and *K. pneumoniae* were isolate from bronchoalveolar lavage fluid.
Not reported	*M. chelonae*	*S. apiospermum*	[126]	Case report in an immunocompetent patient.
anti-IFN-γ ab	*M. intermedium*	*T. marneffei*	[143]	Case report.
Bronchiectasis, anti-IFN-γ ab	*M. fortuitum*	*Aspergillus* sp.	[144]	Case report.
Alcoholics, COPD	*M. avium, M. xenopi*	*Aspergillus* sp.	[142]	Case report.
Miscellaneous conditions (previous *M. xenopi* infection, bronchectasia, cancer)	*M. xenopi*	*Aspergillus* sp.	[157]	Case report.
AIDS	MAC	*H. capsulatum*	[151]	Case report.Disseminated Histoplasma and MAC with recurrence of *Pneumocystis carinii* pneumonia.
AIDS	MAC	*H. capsulatum*	[152]	Case report of disseminated infection (*E. coli* bacteremia).
AIDS	NTM (probably *M. avium-M. intracellulare)*	*H. capsulatum*	[153]	Autopsy Case Report.
Bronchiestasis	MAC	*C. albicans*, *A. fumigatus*	[137]	
Several including MAC pulmonary infection, COPD, bronchiestasis, autoimmune disease, rheumatoid arthritis, corticosteroids use.	MAC	Aspergillus sp.	[138]	Patients with pulmonary MAC disease frequently had chronic coinfection with MSSA, *P. aeruginosa* and Aspergillus.
Lung disease, corticosteroid use	MAC	*A. fumigatus*	[71]	Retrospective monocentric study.
HIV infection	Not specified	*Cryptococcus*	[131]	Prospective study.
Several including COPD and cancer	*M. avium* and *M. intracellulare* (most frequent) *M. abscessus* and *M. gordonae*	*Aspergillus* sp.	[139]	Retrospective study.
Pulmonary disease (e.g., tuberculosis, COPD) and steroid treatment	MAC and MABC	*Aspergillus* spp. (*A fumigatus, A niger, A. terreus, A. flavus*)	[132]	Retrospective study. Only the group infected with NTM that developed chronic pulmonary aspergillosis (CPA) was considered.
Non-cystic fibrosis bronchiectasis with different etiologies, immunosuppression and MAC disease	MAC	*Aspergillus* sp.	[128]	Retrospective study.
Pulmonary MAC disease and several comorbidities (rheumatoid arthritis, steroid use)	MAC	*A. fumigatus*	[127]	Retrospective cohort study.
AIDS	MAC	*H. capsulatum*	[136]	Multicenter Study.
AIDS	NTM	*H. capsulatum*	[155]	Case report.
HIV infection	*M. sherrisii*	*H. capsulatum*	[156]	Case report.
COPD	*M. avium*	*A. fumigatus*	[130]	Review including case report.

Search performed in PubMed database (https://pubmed.ncbi.nlm.nih.gov) using the terms coinfection/ co-infection, immunosuppressed, nontuberculous mycobacteria and fungi. Alternatively, the name of specific NTM and fungi were used as keywords being the search focused on the last 20 years. Ab (antibodies), AIDS (acquired immune deficiency syndrome), HIV (human immunodeficiency virus), MAC (*M. avium* complex), COPD (chronic obstructive pulmonary disease), MSSA (methicillin susceptible *Staphylococcus aureus*), CPA (chronic pulmonary aspergillosis). * Clinical conditions predisposing to NTM infection or likely to affect the immune status causing immunosuppression. ** Previously known as *Penicillium marneffei.*
